# Improving family planning services delivery and uptake: experiences from the “Reversing the Stall in Fertility Decline in Western Kenya Project”

**DOI:** 10.1186/s13104-017-2821-4

**Published:** 2017-10-10

**Authors:** Joshua Amo-Adjei, Michael Mutua, Sherine Athero, Chimaraoke Izugbara, Alex Ezeh

**Affiliations:** 1 African Population and Health Research Center, Nairobi, Kenya; 2 School of Public Health, University of Witwatersrand, Nairobi, Kenya

**Keywords:** Family planning, Services delivery, Partnerships, Africa

## Abstract

**Objective:**

In this paper, we reflect on our experiences of implementing a multipronged intervention to improve sexual and reproductive health outcomes. The project used family planning as its entry point and was implemented in two high fertility counties—Busia and Siaya in Kenya. The intervention, implemented by a seven-member consortium, involved: family planning services delivery; regular training of service providers to deliver high quality services; monitoring and evaluation; strengthening of commodity chain delivery and forecasting; school-based and out-of-school based sexuality education; and advocacy and stakeholder engagements at the community, county and national levels.

**Results:**

Over a 5-year period, the project contributed to raising demand for family planning considerably, evidenced in fertility decline. It also improved the capacity of family planning services providers, increased commitment and awareness of county government and other community stakeholders on the importance of investments in family planning. Our collaborations with organisations interested in sexual and reproductive health issues substantially enhanced the consortium’s ability to increase demand for, and supply of family planning commodities. These collaborations are proving useful in the continuity and sustainability of project achievements.

**Electronic supplementary material:**

The online version of this article (doi:10.1186/s13104-017-2821-4) contains supplementary material, which is available to authorized users.

## Introduction

Family planning (FP) reduces the burden of unplanned pregnancies, promotes smaller families, and improves maternal and child survival, household well-being, and women’s participation in development efforts [1, 2]. These benefits notwithstanding, over 225 million women globally who want to avoid pregnancy do not use effective contraception [3], contributing to 85 million unintended pregnancies [4]. Yet, effective methods to prevent or postpone pregnancy exist.

The drivers of low utilization of family planning services vary across contexts [5, 6]. In sub-Saharan Africa, the major barriers to family planning include pro-natalist values [7], poor livelihoods [8], weak health systems and poorly-trained providers [9], lack of access to quality sexual and reproductive health (SRH) information [10], fears of side effects [11], and weak support by male partners and religious and political authorities [12–15]. In this paper, we briefly describe the triggers to implementing a successful multipronged intervention to improve SRH outcomes, including family planning uptake in two large, generally rural and high-fertility counties in Kenya.

The Reversing the Stall in Fertility Decline in Western Kenya Project was launched in 2009 in Western and Nyanza provinces, which at the time, had the highest average total fertility rate (TFR) of 5.5 against a national TFR of 4.6 in 2008 (see Table 1). Within these two provinces, the project focused on the main districts, Busia and Siaya respectively, both of which became counties following the 2010 Kenyan Constitution.

**Table 1:** Description of fertility rates in Kenya: 1989–2009. Source: 1989; 1993; 1998; 2003; 2008/09 KDHS

Province	Year					Remark
	1989	1993	1998	2003	2009	
Nairobi	4.2	3.4	2.6	2.7	2.8	Stall
Central	6.0	3.9	3.7	3.4	3.4	Stall
Eastern	7.2	5.9	4.7	5.1	4.4	Declining
Rift Valley	7.0	5.7	5.3	5.8	4.7	Declining
Coast	5.4	5.3	5.0	4.9	4.8	Declining
*Nyanza*	*6.9*	*5.8*	*5.0*	*5.6*	*5.4*	*Stall*
*Western*	*8.1*	*6.4*	*5.6*	*5.8*	*5.6*	*Stall*

The goal of the project was to address the unavailability and inaccessibility of family planning services by (1) improving the supply of family planning services at community and facility levels, and (2) increasing the knowledge and demand for family planning services. The project, was implemented by a consortium of seven carefully selected and strategic partners^1^ with longstanding expertise and experience in different aspects of family planning, research, capacity building for providers of family planning services, service delivery, and monitoring and evaluation. Additional file 1 provides descriptions of project partners and their responsibilities. After a series of implementation meetings among partners, the following activities were agreed on for execution.

- Training of FP service providers: project partners conducted regular training for family planning (FP) service providers in both counties. Topics covered in these sessions included provision of short, long-acting, as well as permanent FP methods, including vasectomy; counselling of clients; referral structures/protocols; and commodity logistics management to prevent stock-outs, which is one of the setbacks of FP programs in developing countries [16].
- Family planning service provision: project partners relied on available government infrastructure to provide SRH services, particularly FP in the communities. Marie Stopes Kenya (MSK) and Family Health Options Kenya (FHOK) were the key service delivery partners and used existing government community health volunteers (CHVs) and health facilities to expand access to FP products through community distribution of short-acting methods and referral to health facilities for long-acting permanent methods.
- School-based and out-of-school life-skills education: proven strategies such as edutainment [17], peer education, and use of trained and skilled SRH providers, were s used to reach both in- and out-of-school adolescents and young persons to dispel myths and misconceptions about FP. Sensitization programs were also conducted among adolescents on sexual and reproductive health rights. The work package was overseen by Center for the Study of Adolescence (CSA).
- Community education and mobilization on SRH: MSK and FHOK, working with trained CHVs mobilized community leaders and members furnishing them with information, education and communication on FP. CHVs made presentations on FP at *barazas* 2, women, men and youth group meetings. Other mobilisation activities included the use of social media platforms (e.g. Facebook), project banners, posters, and *talking walls* at schools, branded caps and T-shirts, method bags—to increase visibility of services provided by the project.
- Advocacy on FP and sexuality education with county health and education policymakers: in order to increase recognition and funding of FP and sexuality education continuous advocacy activities were pursued. These were intended to increase political buy-ins and increased funding at both county and national levels. These engagements occurred at quarterly meetings with county and sub-county health management teams. This was important for sustainability given that the project had relatively short lifespan and funded externally.

## Main text

This section focuses on key results recorded under the project. Prior to implementation in 2009, contraceptive prevalence rate (CPR) in Siaya was 33% and in Busia 41% By 2014, CPR for Siaya and Busia stood at 51 and 57% respectively. During the same period, TFR dropped from 5.4 to 4.2 in Siaya, and from 5.6 to 4.7 in Busia. Unmet need for FP also dropped from 32 to 23% and 26 to 21% in Siaya and Busia counties respectively. Table 2 provides a description of the project’s contribution by 2014.

**Table 2:** Changes in fertility and use of FP in Busia and Siaya counties between 2008/9 and 2014

County	TRF		Any CPR		Modern CPR		Unmet need	
	2008/9^a^	2014	2008/9	2014	2008/9	2014	2008/9	2014
Busia	5.6	4.7	47	58	41	57	26	21
Siaya	5.4	4.2	35	55	33	51	32	23
National	4.6	3.9	46	58	39	53	26	18

^a^Data for 2008/2009 was collected at province level, therefore this represents the results for Western (for Busia) and Nyanza (for Siaya) provinces

The project supported the training of 600 (275 in Busia and 325 in Siaya) CHVs on the FP technical module, counselling, referral, and provision of short acting contraceptive methods. A total of 164 health providers, mostly doctors, nurses, and clinical officers were trained on contraceptive technology update (CTU) and FP commodity management skills. An additional 30 community health extension workers (CHEW) were trained on provision of youth-friendly services (YFSs) and equipped with skills to link FP service seekers at the community level to health facilities.

The project reached 213,758 people within the target age groups with FP information through household visits. In addition, 8085 community meetings were held, reaching 402,068 men and women with FP messages. During these two core outreach activities, 78,887 contraceptive pills, 2240 intrauterine contraceptive devices (IUCDs) and 19,745 implants were offered. Other details are presented in Table 3.

**Table 3:** Clients reached and supply of family planning commodities

Family planning method	Service provided	Number of persons
Total clients	Clients new to FP	130,097
	Clients revisiting FP	177,444
	Clients new to project	146,803
	Clients revisiting project	128,068
Pills	Clients new to pills	30,028
	Clients revisiting pills	45,361
	Pills total cycle	185,124
IUCD	IUCD insertion for new clients	863
	IUCD insertion for revisiting clients	1377
	IUCD removals	57
Implant	Implant insertion for new clients	8877
	Implant insertion for revisiting clients	10,868
	Implant removal for clients	767
Bilateral tubal ligation (BTL)	BTL new clients	506
	BTLs for revisiting clients	1128
Vasectomy	Vasectomy for new clients	26
	Vasectomy for revisiting clients	23
Depot medroxyprogesterone acetate (DMPA)	DMPA for new clients	4497
	DMPA for revisiting clients	11,760

The project made some relevant strides in generating the commitment of government towards FP in the counties. Currently, the two county governments have made budgetary allocations to FP. In Siaya County, Ksh 2,000,000 (US$ 20,000) of the health budget was dedicated to FP in the 2015/16 fiscal year, monthly stipend was committed for the payment of CHVs and medical cover was provided for CHVs under the NHIF by the county government. In Busia County, the County Government made allocations in the 2015/2016 financial year to support CHVs to initiate income-generating activities, and indications were that monthly stipends for CHVs would be captured in the 2016/2017 budget. In 2015, the county also launched an FP/RH strategic plan supported by CSA. In the following paragraphs, we highlight some key learnings and recommendations for similar interventions in comparable contexts.

First, building the capacity of service providers on FP commodities management contributed positively in preventing or minimising stock-outs and ensuring the availability of services/products. Second, the use of CHVs and CHEWs offered an opportunity to cover a wider geographic space, allowing partners to distribute more commodities and to refer more people for services. Also, by building the capacity of CHVs, the quality of routine data generated was appreciably high. CHVs also made it possible to deliver short-acting methods and offer counselling on LAPM to community members.

Another important lesson relates to the benefits of integrating FP services into other reproductive health (RH) services. In the project, we included cervical cancer screening and other services in what we offered which helped to attract people to the project. Similar interventions could benefit by integrating other RH services into family planning demand generation activities.

The long-held view about the importance of male-involvement in FP [18, 19] was demonstrated in the areas where we operated. Men using different modern FP methods or whose partners were using FP methods served as change agents in motivating other men to support or use FP methods particularly vasectomy. In settings such as ours, the role of men is indispensable in FP adoption and they should be constantly engaged in family planning work.

The need to work with, strengthen, and support existing government structures was another key lesson from this intervention. With national MoH serving as external quality assurance team, services delivered were consistent with national FP standards. Thus, similar projects in comparable contexts must be anchored in the existing health management and health financing frameworks for improved sustainability measures.

In conclusion, working with organisations and groups with shared interests substantially enhanced the consortium’s ability to engage with government and other gatekeepers in driving the project’s goals. Also worth mentioning is the cost-effectiveness and strong in-built accountability mechanisms this approach offers. The consortium approach allowed partners to capitalise on the abilities and strengths of each other, generate trust among community members and ultimately, reaching out to communities, with little duplication of efforts.

## Limitations

The project was externally funded, creating problems of sustainability. Funding is a persistent challenge to sustained FP programming in Kenya [20] and many other developing countries in sub-Saharan Africa [21]. Despite the immense contributions of CHVs to the project, the low level of education among the majority coupled with multi-tasking and sometimes “fragmented” focus of CHVs [22] (concurrently involved in other health interventions) constrained their ability to focus on the project.

Myths and misconceptions about FP and SRH services (e.g. likening vasectomy to castration) and fears of contraceptive failure among HIV-infected women who are on anti-retroviral treatment/therapy (ART) still exist in the communities.

High staff turnover due to frequent transfers, especially of those who had been trained on LAPM was a recurrent strain on the project. This created skill-gaps and sometimes led to interruptions in service delivery. Many CHVs also left their employment in pursuit of other interests.

The 2013 devolution which transferred health functions from national to county governments also resulted in new priorities by the county administrations, rather than the priorities previously set by the national MoH. This called for adjustments within project partners in order to align interventions with new county priorities.

## Additional file

**Additional file 1.** Title of data: Description of project partners and their roles. Description of data: Project partners and their roles and responsibilities.

## Abbreviations

APHRC: African Population and Health Research Center
ASRH: adolescent sexual and reproductive health
CHAK: Christian Health Association of Kenya
CHEW: community health extension worker
CHV: community health volunteer
CSA: center for the study of adolescence
FAWE-K: Forum for African Women Educationalist-Kenya
FP: family planning
IEC: information, education and communication
LAPM: long-acting and permanent (contraceptive) methods
MSK: Marie Stopes Kenya
SACs: short-acting contraceptive methods
SRH: sexual and reproductive health

## Footnotes

1 African Population and Health Research Center (APHRC), Great Lakes University of Kisumu (GLUK), Marie Stopes Kenya (MSK), Family Options Kenya (FHOK), center for the study of adolescence (CSA), Forum for African Women Educationalist—Kenya (FAWE-K), Christian Health Association of Kenya (CHAK).

2 Swahili word for a deliberation meeting held by a collective group of people. It is often used on public gatherings called by local public administrative officers such as chiefs to address their communities over emerging issues of administrative interest. Many or (some) of these meetings are mandatory and programs implementers find these meetings an easy access to majority of the community, while gaining support and buy-in from the public administration, as well as civic and local political leadership.

## References

[1] Denton EH. Benefits of family planning. In: McFarlane DR, editor. Global Population and Reproductive Health. Burlington: Jones and Bartlet Learning; 2014. p.199–221.

[2] Chola L, McGee S, Tugendhaft A, Buchmann E, Hofman K (2015). Scaling up family planning to reduce maternal and child mortality: the potential costs and benefits of modern contraceptive use in South Africa. PLoS ONE.

[3] United Nations Population Fund. Family planning—overview. UNFPA; 2016.

[4] Sedgh G, Singh S, Hussain R (2014). Intended and unintended pregnancies worldwide in 2012 and recent trends. Stud Fam Plan.

[5] Jain AK, Ross JA (2012). Fertility differences among developing countries: are they still related to family planning program efforts and social settings?. Int Perspect Sex Reprod Health.

[6] Ali M, Seuc A, Rahimi A, Festin M, Temmerman M (2014). A global research agenda for family planning: results of an exercise for setting research priorities. Bull World Health Organ.

[7] Nalwadda G, Mirembe F, Byamugisha J, Faxelid E (2010). Persistent high fertility in Uganda: young people recount obstacles and enabling factors to use of contraceptives. BMC Public Health.

[8] Speizer IS, Nanda P, Achyut P, Pillai G, Guilkey DK (2012). Family planning use among urban poor women from six cities of Uttar Pradesh, India. J Urban Health.

[9] Jongh T, Gurol-Urganci I, Allen E, Jiayue Zhu N, Atun R (2016). Barriers and enablers to integrating maternal and child health services to antenatal care in low and middle income countries. BJOG Int J Obstet Gynaecol.

[10] Alkema L, Kantorova V, Menozzi C, Biddlecom A (2013). National, regional, and global rates and trends in contraceptive prevalence and unmet need for family planning between 1990 and 2015: a systematic and comprehensive analysis. Lancet.

[11] Mosha I, Ruben R, Kakoko D (2013). Family planning decisions, perceptions and gender dynamics among couples in Mwanza, Tanzania: a qualitative study. BMC Public Health.

[12] Bawah AA, Akweongo P, Simmons R, Phillips JF (1999). Women’s fears and men’s anxieties: the impact of family planning on gender relations in northern Ghana. Stud Fam Plan.

[13] Bongaarts J (2014). The impact of family planning programs on unmet need and demand for contraception. Stud Fam Plan.

[14] Withers M, Dworkin SL, Zakaras JM, Onono M, Oyier B, Cohen CR, Bukusi EA, Grossman D, Newmann SJ (2015). **‘**Women now wear trousers’: men’s perceptions of family planning in the context of changing gender relations in western Kenya. Cult Health Sex.

[15] Gammeltoft T (2012). Women’s bodies, women’s worries: health and family planning in a Vietnamese rural commune.

[16] Hancock NL, Vwalika B, Sitali ES, Mbwili-Muleya C, Chi BH, Stuart GS (2015). Evaluation of service quality in family planning clinics in Lusaka, Zambia. Contraception.

[17] Keyonzo N, Nyachae P, Kagwe P, Kilonzo M, Mumba F, Owino K, Kichamu G, Kigen B, Fajans P, Ghiron L (2015). From project to program: Tupange’s experience with scaling up family planning interventions in urban Kenya. Reprod Health Matters.

[18] Theuring S, Mbezi P, Luvanda H, Jordan-Harder B, Kunz A, Harms G (2009). Male involvement in PMTCT services in Mbeya Region, Tanzania. AIDS Behav.

[19] Mwaikambo L, Speizer IS, Schurmann A, Morgan G, Fikree F (2011). What works in family planning interventions: a systematic review. Stud Fam Plan.

[20] Sonalkar S, Mody S, Phillips S, Gaffield ME (2013). Programmatic aspects of postpartum family planning in developing countries: a qualitative analysis of key informant interviews in Kenya and Ethiopia. Afr J Reprod Health.

[21] Ezeh A (2016). Population’s part in mitigating climate change: a Nigerian response. Bull At Sci.

[22] Tulenko K, Mgedal S, Afzal MM, Frymus D, Oshin A, Pate M, Quain E, Pinel A, Wynd S, Zodpey S (2013). Community health workers for universal health-care coverage: from fragmentation to synergy. Bull World Health Organ.

