# Supplementary material for: Improving family planning services delivery and uptake: experiences from the “Reversing the Stall in Fertility Decline in Western Kenya Project”
Source: BMC Res Notes. 2017 Oct 10;10:498. doi: 10.1186/s13104-017-2821-4 (PMC5634860; doi:10.1186/s13104-017-2821-4)
Supplement: Supplementary file 1 — Additional file 1. Title of data: Description of project partners and their roles. Description of data: Project partners and their roles and responsibilities. [file 13104_2017_2821_MOESM1_ESM.docx]

**Title of data: Description of project partners and their roles**

**Description of data: Project partners and their roles and responsibilities**

1. *African Population and Health Research Centre (APHRC)*

A well-established research institute with long-standing reputation for high-quality impact research in sub-Saharan Africa, APHRC was founded in 2001. The institute actively uses evidence to engage policymakers and other key stakeholders to achieve measurable policy impacts and ensure decision making is informed by rigorous research. APHRC was the lead partner on the Reversing the Stall in Fertility Decline in Western Kenya Project. It also provided research, monitoring, learning and evaluation support to the entire project.

1. *Great Lakes University of Kisumu (GLUK)*

GLUK is one of the Kenya’s leading universities in tropical health research with emphasis on community health. The university succeeded the Tropical Institute of Community Health and Development (TICH) in Africa in 1998. It was inspired by a need offer tertiary academic courses in Community Based Health Care. Working closely with the lead partner, APHRC, GLUK was responsible for monitoring and evaluation of project activities.

1. *Marie Stopes, Kenya (MSK)*

Since 1985 when Marie Stopes was established in Kenya, it has, in collaboration with the government of Kenya, spearheaded delivery of high-quality and client-centred reproductive health services to women, men and young people in the country. MSK is a leader in the delivery of quality reproductive health clinical services, mobile family planning services, and social franchising for health. On the project, MSK provided community RH education and mobilization, FP service delivery, and training of RH services providers. It also supported advocacy and policy engagement activities.

1. *Family Health Options Kenya (FHOK).*

FHOK has over 50 years’ experience in providing sexual and reproductive health services in Kenya. Apart from delivering comprehensive and quality reproductive health services, it is a leader in capacity building on sexual and reproductive health matters, gender mainstreaming, and youth empowerment and other areas of expertise. Together with MSK, FHOK provided community RH education and mobilization, FP service delivery, and training of RH services providers. It also supported advocacy ad policy engagement activities.

1. *Centre for the Study of Adolescence (CSA)*

CSA has operated in Kenya since 1988. The organization is driven by a desire to improve adolescent health through research, advocacy, and implementation of programmes and interventions geared towards advancing adolescent health in Kenya. CSA’ role in the Project was providing comprehensive sexuality education to in- and out-of-school young people from 10-24 years.

1. *Forum for African Women Educationalist-Kenya (FAWE-K)*

FAWE, which has worked in Kenya since 1992, seeks to exploit the potentials of women in Africa for socioeconomic transformation. This vision is achieved through policy and community advocacy and the development and promotion of contexts that are conducive to girls’ enrolment, continuation and successful completion of the school cycle. On the project, FAWE trained women and girls, teachers, and parents on the benefits of FP in keeping girls in school and also provided support to school girls who become pregnant to return to school. It also provided sexuality education to in-school youth and supported advocacy at policy and community levels.

1. *Christian Health Association of Kenya (CHAK)*

From inception in 1930s as Hospitals Committee of the National Council of Churches (NCCK), CHAK has evolved into a leader in facilitating the role of religious institutions in the delivery of FP services and promotion of reproductive health in Kenya. The main role of CHAK in the Reversing the Stall in Fertility Decline in Western Kenya Project was the engagement and training of religious leaders on FP messaging ,service provision, and policy advocacy.

The project was implemented in close collaboration with the Ministry of Health – Government of Kenya. Through the Division of Reproductive Health (DRH) and the Division of Community Health Services (DCHS), the Ministry of Health provided supervisory oversight for the Project. This ensured that SRH services provided by partners met national standards. The two divisions were also critical in communicating lessons from the project to health policy makers in government.
